# Glucocorticoid-Induced Osteoporosis in Children with 21-Hydroxylase Deficiency

**DOI:** 10.1155/2013/250462

**Published:** 2013-01-08

**Authors:** Annamaria Ventura, Giacomina Brunetti, Silvia Colucci, Angela Oranger, Filomena Ladisa, Luciano Cavallo, Maria Grano, Maria Felicia Faienza

**Affiliations:** ^1^Department of Biomedical Sciences and Human Oncology, University of Bari, Piazza G. Cesare 11, 70124 Bari, Italy; ^2^Section of Human Anatomy and Histology, Department of SMB-NOS, University of Bari, 70124 Bari, Italy

## Abstract

21-Hydroxylase deficiency (21-OHD) is the most common cause of congenital adrenal hyperplasia (CAH), resulting from deletions or mutations of the P450 21-hydroxylase gene (*CYP21A2*). Children with 21-OHD need chronic glucocorticoid (cGC) therapy, both to replace congenital deficit in cortisol synthesis and to reduce androgen secretion by adrenal cortex. GC-induced osteoporosis (GIO) is the most common form of secondary osteoporosis that results in an early, transient increase in bone resorption accompanied by a decrease in bone formation, maintained for the duration of GC therapy. Despite the conflicting results in the literature about the bone status on GC-treated patients with 21-OHD, many reports consider these subjects to be at risk for osteoporosis and fractures. In bone cells, at the molecular level, GCs regulate various functions including osteoblastogenesis, osteoclastogenesis, and the apoptosis of osteoblasts and osteocytes. In this paper, we focus on the physiology and biosynthesis of endogenous steroid hormones as well as on the effects of GCs on bone cells, highlighting the pathogenetic mechanism of GIO in children with 21-OHD.

## 1. Introduction

21-Hydroxylase deficiency (21-OHD) is the most common cause of congenital adrenal hyperplasia (CAH), caused by sequence variants in the 21-hydroxylase gene (*CYP21A2*) [1]. This disorder is characterized by accumulation of the precursors immediately proximal to the 21-hydroxylation step along the pathway of cortisol synthesis, which are shunted into the androgen pathway. Children with 21-OHD need chronic glucocorticoid (cGC) therapy as soon as they are diagnosed with the disease, both to correct the deficiency in cortisol and to reduce androgen secretion by adrenal cortex [2]. 

An organ system that has the potential to be profoundly affected by cGC therapy is the skeleton, and GC-induced osteoporosis (GIO) is the most common form of secondary osteoporosis [3]. GIO results in an early, transient increase in bone resorption accompanied by a decrease in bone formation, which is maintained for the duration of GC therapy. Although many patients remain asymptomatic, fractures occur in 30–50% of GCs-treated patients [4].

Recently, several studies have helped to clarify the mechanisms responsible for GIO, highlighting the molecular events occurring in skeletal cells.

Three principal cell types are involved in bone modeling and remodeling: osteoblasts (OBs), osteoclasts (OCs), and osteocytes, each with distinct and varying functions. The actions of these cells are modulated and coordinated by autocrine, paracrine, and endocrine regulators, such as cytokines, growth factors, and hormones. In bone cells, at the molecular level, GCs regulate various functions including osteoblastogenesis, osteoclastogenesis, and the apoptosis of osteoblasts and osteocytes [5].

In this paper, we focus on the physiology and biosynthesis of endogenous steroid hormones as well as on the effects of GCs on bone cells, highlighting the pathogenetic mechanism of GIO in children with 21-OHD.

## 2. Physiology and Biosynthesis of Steroid Hormones

Steroid hormones serve many essential roles in mammalian physiology, ranging from promoting development to regulation of metabolism. Two of the major steroidogenic tissues in mammals include the adrenal glands and gonads [6].

Based on its functional actions, steroid hormones are classified into five principal classes: estrogens (estradiol, estrone, and estriol), progestins (progesterone), androgens (testosterone, A4, and dihydrotestosterone), glucocorticoids (cortisol, corticosterone), and mineralcorticoids (aldosterone, deoxycorticosterone) [7].

The main adrenal steroids that enter the circulation are aldosterone, which is important in salt homeostasis and acid excretion; cortisol, which is involved in a range of homeostatic processes including carbohydrate, protein, and fat metabolism and regulation of immune processes; dehydroepiandrosterone (DHEA) and androstenedione, the primary source of circulating androgens in women [8].

Cortisol and adrenal androgen production are regulated by the hypothalamic-pituitary-adrenal (HPA) axis. The production of corticotropin releasing hormone (CRH) by the hypothalamus stimulates adrenocorticotropic hormone (ACTH) release by the anterior pituitary gland which in turn stimulates the synthesis of cortisol by the adrenal cortex.

**Figure 1:**
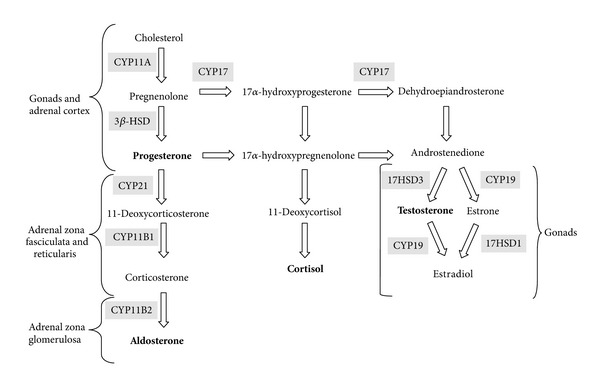
Biosynthesis of steroid hormones in adrenal glands and gonads. Enzymes are highlighted. Final steroid hormone product is in bold.

All steroid hormones are derived from cholesterol through a complex series of chemical modifications [9].  Figure 1 shows the biosynthesis of steroid hormones in adrenal glands and gonads.

The rate-limiting step in steroid biosynthesis is importation of cholesterol from cellular stores to the matrix side of the mitochondria inner membrane. The first enzymatic step in steroid synthesis is the conversion of cholesterol, a C27 compound, to the C21 steroid pregnenolone [10]. This is catalyzed by the mitochondrial cytochrome P450 enzyme CYP11A. Pregnenolone is the common precursor for all other steroids and, as such, may undergo metabolism by several other enzymes. To synthesize mineralocorticoids, 3*β*-hydroxysteroid dehydrogenase (3*β*-HSD) in the endoplasmic reticulum and mitochondria converts pregnenolone to progesterone. This is 21-hydroxylated in the endoplasmic reticulum by CYP21A2 to produce deoxycorticosterone (DOC). Aldosterone is produced by the 11 *β*-hydroxylation of DOC to corticosterone, followed by 18-hydroxylation and 18-oxidation of corticosterone by CYP11B2 enzyme. To produce cortisol, the major glucocorticoid in man, CYP17 converts pregnenolone to 17*α*-hydroxypregnenolone [11]. 3*β*-HSD utilizes 17*α*-hydroxypregnenolone as a substrate, producing 17*α*-hydroxyprogesterone. The latter is 21-hydroxylated by CYP21A2 to form 11-deoxycortisol, which is converted to cortisol by CYP11B1 in mitochondria. The 17,20-lyase activity of CYP17 converts 17*α*-hydroxypregnenolone to dehydroepiandrosterone (DHEA, a C19 steroid, and sex hormone precursor). DHEA is further converted by 3*β*-HSD to androstenedione. In the gonads, this is reduced by 17*β*-hydroxysteroid dehydrogenase to testosterone. In pubertal ovaries, aromatase (CYP19) can convert androstenedione and testosterone to estrone and estradiol, respectively. Testosterone may be further metabolized to dihydrotestosterone by steroid 5*α*-reductase in androgen target tissues [9].

## 3. Abnormal Steroids in 21-Hydroxylase Deficiency

Inefficient cortisol synthesis in 21-OHD patients signals the anterior pituitary to increase ACTH release, with subsequent overstimulation and hyperplasia of the adrenals. 

Rather than cortisol and aldosterone, the adrenals produce excess of sex hormone precursors that are further metabolized to active androgens (testosterone and dihydrotestosterone) and to a lesser extent estrogens (estrone and estradiol) [12].

The most definitive hormonal diagnostic test for 21-OHD is an ACTH-stimulation test, which measures the serum concentrations of 17*α*-hydroxyprogesterone, the main substrate for 21-hidroxylase, at 0 and 60 min after ACTH administration [13].

Three forms of 21-OHD can be distinguished by means of clinical, hormonal, and molecular-genetic criteria: the classical salt wasting (SW), classical simple virilizing (SV), and nonclassical forms (NC). In SW-CAH, affected children present with salt loss during the neonatal period, and females foetuses will develop virilizing malformations of external genitalia. Patients with SV-CAH do not develop life-threatening salt loss, but female newborns present virilized genitalia, and boys may develop precocious pseudopuberty during early childhood. NC-CAH is characterized by various degrees of late-onset symptoms. The most common symptoms are premature pubarche in children, acne, hirsutism, and menstrual irregularities in young women [14].

Children with 21-OHD need chronic cGC therapy as soon as they are diagnosed with the disease in order to reduce excessive ACTH and consequent increase androgen production, by substituting for deficient cortisol and when necessary mineralocorticoid synthesis [15].

During childhood, the main aims of the medical treatment of CAH are to prevent salt loss and virilization, to achieve normal stature and to undergo normal puberty [16].

Undertreatment exposes the patient to the risk of adrenal crisis and allows increased adrenal androgen production, with consequent advancement of bone age and loss of growth potential. Overtreatment, however, results in growth retardation, truncal obesity, and osteopaenia, through the effects of steroids on growth hormone secretion and bone metabolism [15].

Hydrocortisone (HC) is considered the drug of first choice in CAH during infancy and childhood [17].

## 4. Molecular Genetics of 21-OHD

The gene encoding 21-hydroxylase, *CYP21A2*, is located in the HLA region III on the short arm of chromosome 6 (6p21.3) closely linked to a nonfunctional pseudogene *CYP21A1P *[1]. Both genes consist of 10 exons sharing a high degree of homology with a nucleotide identity of 98% on exon and of 96% on intron level [1]. The high homology of these regions causes misalignment during meiosis, resulting in intergenic recombinations that are responsible for 95% of the mutations associated with 21-OHD; the remaining 5% of mutations appear to be the result of spontaneous mutations rather than gene conversion events [18]. 

Approximately 95% of all inactivating mutations of *CYP21A2* comprise deletions/large gene conversions of the entire gene and/or a few point mutations [12].

NC and classical forms of 21-OHD are associated with distinct genotypes, characterized by varying levels of enzyme activity. The genotype for the classical form of 21-OHD is predicted to be a severe mutation on both alleles at the 21-hydroxylase locus, with markedly reduced enzymatic activity generally associated with SW. Patients with NC form of 21-OHD are predicted to have mild mutations on both alleles, or one severe and one mild mutation of *CYP21A2* (compound heterozygotes) [13]. A good genotype-phenotype correlation has been shown in 98% of 21-OHD patients; however, rare cases of nonconcordance have important implications in prenatal diagnosis of 21-OHD and genetic counseling [13].

The Endocrine Society Clinical Practice Guidelines from 2010 recommends genotyping for purposes of genetic counseling and for confirmation of the diagnosis especially in NC-CAH when the ACTH-stimulation test is equivocal [17].

## 5. Molecular Effects of GCs on Bone Cells

### 5.1. Osteoblasts

The reduction in OB number and function has a central role in the pathogenesis of GIO, leading to a suppression of bone formation characteristic of GCs excess. The mechanism includes inhibition of replication and differentiation and enhanced apoptosis of OBs [19, 20]. 

GCs decrease the replication of osteoblastic lineage cells, reducing the pool of cells that may differentiate into mature OBs [5]. 

In the presence of GCs, bone marrow stromal cells differentiation is redirected towards adipocyte lineage. Mechanisms involved include the induction of peroxisome proliferator-activated receptor *γ*2 (PPAR*γ*) and the regulation of nuclear factors of the CAAT enhancer-binding protein family (C/EBPs), adipocyte P2, aP2; the differentiation-dependent adipocyte protein is a downstream target gene of PPAR*γ* and C/EBP*α* [21] abundantly expressed in the cytoplasm and nuclear region of adipocytes [22]. PPAR*γ* and C/EBP*α* might also indirectly reduce OBs proliferation, decreasing IGF-I transcription [19].

An additional effect of GCs is represented by inhibition of Wnt-*β*-catenin signaling [19], a key pathway for promoting osteoblastogenesis. GCs suppress the canonical Wnt-*β*-catenin signalling pathway in OBs, enhancing the expression of Dickkopf-1 (DKK1), an extracellular Wnt inhibitor which prevents Wnt binding to its receptor complex, and destabilizing *β*-catenin via activation of glycogen synthase kinase 3-b [23, 24]. Moreover, GCs inhibit OB differentiation through the repression of bone morphogenetic protein 2 (BMP2), which has a key role in bone formation [25, 26].

GCs impair the function of the differentiated mature cells, inhibiting OB-driven synthesis of type I collagen (by transcriptional and posttranscriptional mechanisms) [27], the major component of the bone extracellular matrix, with a consequent decrease in bone matrix available for mineralization [19]. 

Moreover, GCs modify osteocalcin gene expression via the GC-responsive elements, which have been identified in the osteocalcin promoter [28, 29].

The proapoptotic effects of GCs on OBs are explicated by modulating the expression of proapoptotic and antiapoptotic genes, such as *BCL2, BIRC5, *and* BCL2L11* [30, 31]. O'Brien et al. demonstrated the requirement of GC signaling in late-stage differentiation of OBs for apoptosis *in vivo* [20]. Dexamethasone (Dex) induction of the protein Bim, a proapoptotic Bcl-2 family member, enhances the activities of its downstream effectors, caspases -3, -7, and -8, and has been suggested as a key regulator of glucocorticoid receptor-dependent OB apoptosis [32].

### 5.2. Osteocytes

The loss of osteocytes might be particularly important in terms of bone structure because these mechanosensors are essential in the repair of bone microdamage. Loss of osteocytes might disrupt the osteocyte–canalicular network, resulting in a failure to detect signals that normally stimulate the replacement of damaged bone. GCs affect the function of osteocytes, by modifying the elastic modulus surrounding osteocytic lacunae. As a result, the normal maintenance of bone through this mechanism is impaired, and the biomechanical properties of bone are compromised [33]. Another direct effect of GCs on osteocytes is the induction of apoptosis through activation of caspase 3 [34].

### 5.3. Osteoclasts

The initial bone loss occurring in patients exposed to GCs might be secondary to increased bone resorption by OCs [3].

OCs are members of the monocyte-macrophage family, derived from the fusion of marrow-derived mononuclear phagocyte, the OC precursors (OCPs), which circulate in peripheral blood (PB) [35]. These cells differentiate under the influence of two cytokines, namely macrophage colony-stimulating factor (M-CSF) and receptor activator of nuclear factor k-B ligand (RANKL). RANKL expressed on OBs and stromal cells as a membrane-bound protein and cleaved into a soluble molecule (sRANKL) by metalloproteinase [36] promotes differentiation and fusion of OCPs and activates mature OCs to reabsorb bone by binding to its specific receptor RANK. Osteoprotegerin (OPG), a soluble decoy receptor secreted by OBs and bone marrow stromal cells, competes with RANK in binding to RANKL, preventing its osteoclastogenic effect [36].

GCs increase the expression of RANKL and decrease the expression of OPG in stromal cells and OBs [37]. GCs also enhance the expression of M-CSF, which in the presence of RANKL induces osteoclastogenesis [37]. Moreover, GCs have been demonstrated to upregulate receptor subunits for osteoclastogenic cytokines of the glycoprotein 130 family [38]. In a work by Takuma et al. [39] are explained the effects of GCs on OC formation. In particular, this study demonstrated that Dex downregulates endogenous interferon-*β* production, an autocrine cytokine that normally inhibits OCs differentiation, allowing osteoclast progenitors to be freed from its differentiation-depressing effect and to proceed toward the phenotype of mature OCs.

## 6. Glucocorticoid Receptor-Mediated Effect ****of GCs 

The GC-induced effects described above appear to be dependent on the duration and concentration of GC treatment and possibly on the differentiation stage of bone cells [4, 40], while data on the exact role of glucocorticoid receptor (GR) in mediating GCs actions are limited.

GR is a ligand-regulated transcription factor, a member of the nuclear-receptor (NR) superfamily that controls gene expression linked to several processes like inflammation, stress responses, glucose homeostasis, lipid metabolism, proliferation, and apoptosis development [41]. In the absence of ligand, GR is associated to the hsp90 chaperone heterocomplex and primarily localizes in the cytoplasm, while the GR-ligand complex is mainly nuclear. In the nucleus, the activated GR regulates gene expression through two modes of action [42, 43]. A direct mechanism involves GR homodimer binding to positive or negative glucocorticoid response elements (GREs) located in the promoter region of target genes, leading to transcription activation or repression, respectively. The activated GR may also function through an indirect mechanism by interacting as a monomer with other transcriptional factors, such as NF-kB or AP-1 [44], without direct binding to DNA. Both GR modes of action would be independent, and it has been postulated that GC beneficial effects (immunosuppressant and anti-inflammatory effects) are associated to the indirect-transrepression mechanism, while the side effects are associated to the direct transactivation one. 

Therefore, extensive efforts are aimed at developing selective GR agonists (SEGRAs) as novel therapies with an improved risk/benefit ratio. The concept of SEGRAs is based on the fact that they largely mediate their effects via transrepression by GR monomers and not through transactivation by GR dimers. Moreover, SEGRAs will serve as a tool to further investigate the molecular basis of GC side effects.

Compound A (CpdA), a plant-derived phenyl aziridine precursor, is a well-investigated agent that mediates its effects by binding the GR [45]. In a recent work, Thiele et al. [46] assessed the effects of CpdA on bone metabolism in a mouse model of GIO. In particular, they examine the effects on the skeleton of CpdA and prednisolone (PRED) using quantitative computed tomography, bone histomorphometry, serum markers of bone turnover, and gene expression analysis. Mice treated with PRED showed a reduction of the total and trabecular bone density in the femur and in the spine, increase of osteoclast number, serum CTX-1 and the skeletal RANKL/OPG ratio, reduced skeletal expression of osteoblast markers, and increased serum levels of DKK-1. None of these effects were observed with CpdA, and consistent with the *in vivo *data, CpdA did not increase the RANKL/OPG ratio in MLO-Y4 cells and failed to transactivate DKK-1 expression in bone tissue, BMSCs, and osteocytes. This study underlines the bone-sparing potential of CpdA and confirms that GC enhanced DKK1 and RANKL expression significantly, in accordance with previous studies.

## 7. Pathogenetic Mechanism of GIO in Children with 21-OHD

Previous reports on 21-OHD patients showed increased [47], decreased [48–57], or normal bone mineral density (BMD) [58–62].

These contradictory results may be explained by heterogeneous populations and methods, as the reports differ with respect to age selections and GC regimens [15]. cGC therapy is known to generate bone loss in many ways: a direct suppression of osteoblastic activity [63] and an inhibition of digestive calcium absorption with secondary hyperparathyroidism and increased bone resorption by osteoclasts [64]. Two studies have evaluated fractures in CAH patients [56, 65]. The study by Falhammar et al. [56] included 61 women with 21-OHD and 61 age-matched women as controls. Results indicated a higher frequency of fractures in women with CAH. When only osteoporotic fractures (vertebrae, wrist, and hip) were considered, the difference almost reached significance (*P* = 0.058). This is of importance for CAH patients, even if this finding has to be confirmed in larger studies, which should evaluate differences in lifestyle between patients and controls, as the trauma leading to fractures was not ascertained. The second study [65] reported vertebral compression fractures in a young adult male with 21-OHD, the onset of which likely corresponds to excessive GC dosing during adolescence.

Biochemical markers of bone turnover have been partially evaluated in patients with CAH [50, 52, 55, 56, 58], and the literature data are inconclusive. Bone turnover was found to be lower in patients with CAH than in controls, and osteocalcin levels correlated positively with growth velocity and negatively with BMD [50, 58]. Another study showed higher bone-specific alkaline phosphatase (ALP) and serum **β**-C-telopeptide of type I collagen (CTX) concentrations in young CAH patients compared with control subjects [55]. In the report of Falhammar et al. [56], the bone resorption marker CTX was found to be reduced in the older group of patients both compared with controls and younger patients. This was not in accordance with the findings of Sciannamblo et al. [55] and Zimmermann et al. [57] that observed elevated CTX concentrations in young individuals, some who are still growing. The authors concluded that the CAH patients treated for many years had predominantly low bone formation but also unexplained low bone resorption [56]. 

Faienza et al. [66] demonstrated a high osteoclastogenic potential of peripheral blood mononuclear cells (PBMCs) in children with 21-OHD on long-term GC treatment. In particular, spontaneous osteoclastogenesis, without adding MCSF and RANKL, and significantly higher osteoclasts resorption activity occurred in 21-OHD patients. Conversely, MCSF and RANKL were essential to trigger and sustain osteoclastogenesis in controls. This spontaneous osteoclastogenesis seems to be supported by both the presence of circulating OCPs and factors released by T cells. In particular, Faienza et al. identified a significant percentage of CD11b-CD51/CD61- and CD51/61-RANK-positive cells, which are OCPs strongly committed. Moreover, evidences supporting a T cell regulation of osteoclastogenesis came from 21-OHD patients' T-cell-depleted PBMC cultures, in which the addition of exogenous M-CSF and RANKL was necessary for OC formation. In fact, T-cells from 21-OHD patients expressed high levels of RANKL and low levels of OPG with respect to controls. Furthermore, 21-OHD patients had higher soluble RANKL and lower OPG serum levels compared with controls. Moreover, we, very recently, demonstrated high DKK1 levels in sera and circulating monocytes, T lymphocytes, and neutrophils from 21-OHD patients [67]. The serum from patients containing elevated levels of DKK1 can directly inhibit osteoblast differentiation *in vitro* as well as affect the expression of RANKL in osteoblasts [66]. We also found a correlation between both DKK1 and RANKL or CTX serum levels in patients. Thus, chronic GC treatment in 21-OHD patients may contribute both to the alteration of bone resorption and formation [66, 67].

## 8. Conclusions

Despite the conflicting results in the literature about the bone status on GC-treated patients with 21-OHD, many reports consider these subjects to be at risk for osteoporosis and fractures. Furthermore, it should be a useful monitoring bone status in treated 21-OHD children, checking BMD and bone turnover markers, in order to avoid GIO in adulthood.

Other studys should be performed to analyze the expression of regulators of bone resorption and bone formation in 21-OHD patients.
